# Supplementary figures and images for: The Impact of Bamboo Consumption on the Spread of Antibiotic Resistance Genes in Giant Pandas
Source: Vet Sci. 2023 Oct 24;10(11):630. doi: 10.3390/vetsci10110630 (PMC10675626; doi:10.3390/vetsci10110630)

SHOOT LEAF CULM

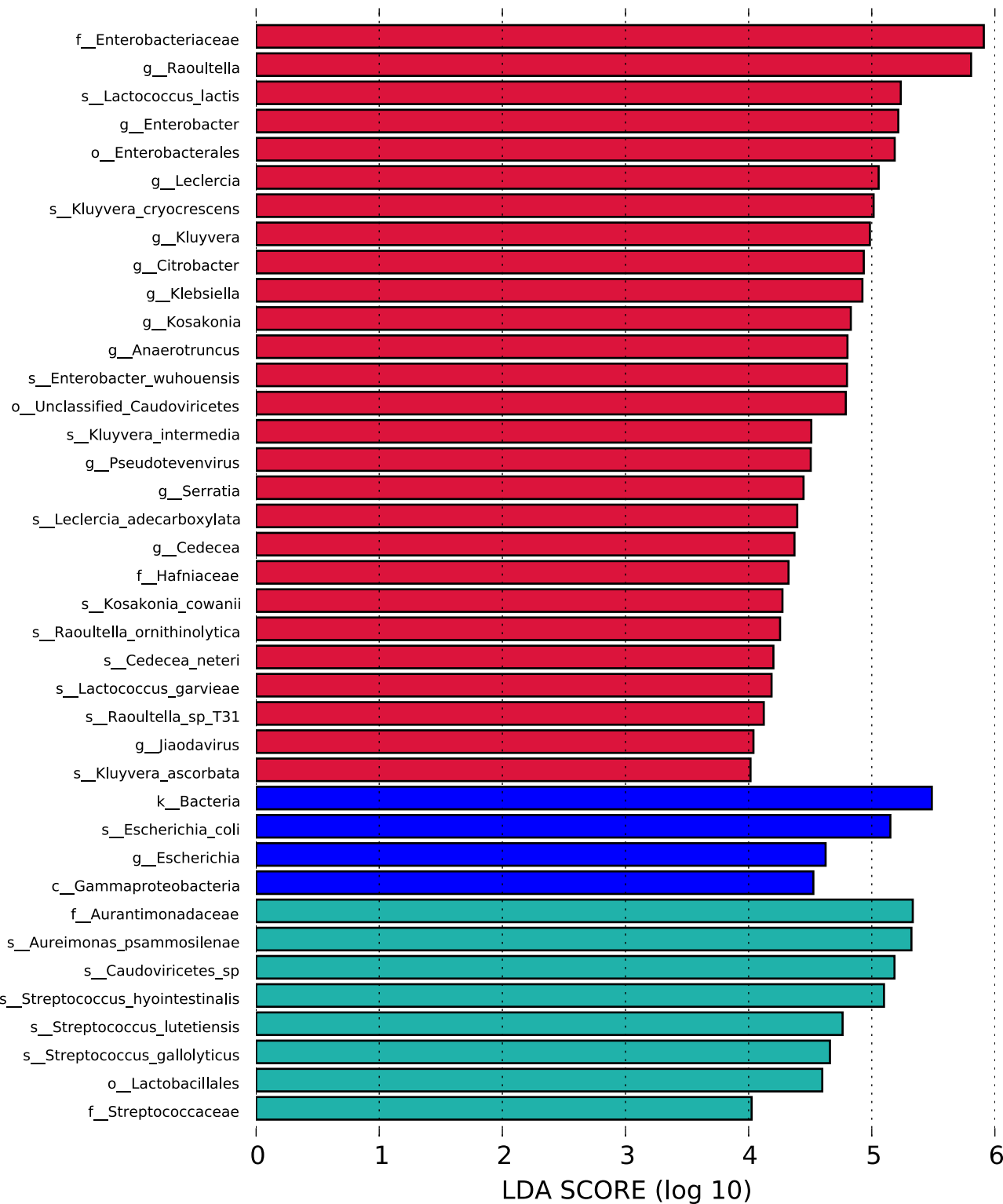

Supplement: Supplementary file 1 [file vetsci-10-00630-s001.zip › vetsci-2616862-supplementary.pdf]
